# Spatial Genetic Structure of the Abundant and Widespread Peatmoss *Sphagnum magellanicum* Brid.

**DOI:** 10.1371/journal.pone.0148447

**Published:** 2016-02-09

**Authors:** Magni Olsen Kyrkjeeide, Kristian Hassel, Kjell Ivar Flatberg, A. Jonathan Shaw, Narjes Yousefi, Hans K. Stenøien

**Affiliations:** 1 NTNU University Museum, Norwegian University of Science and Technology, NO-7491, Trondheim, Norway; 2 Norwegian Institute for Nature Research, N-7485, Trondheim, Norway; 3 Duke University, Department of Biology, Durham, North Carolina, 27708, United States of America; 4 Centre for Biodiversity Dynamics, NTNU University Museum, Norwegian University of Science and Technology, NO-7491, Trondheim, Norway; Field Museum of Natural History, UNITED STATES

## Abstract

Spore-producing organisms have small dispersal units enabling them to become widespread across continents. However, barriers to gene flow and cryptic speciation may exist. The common, haploid peatmoss *Sphagnum magellanicum* occurs in both the Northern and Southern hemisphere, and is commonly used as a model in studies of peatland ecology and peatmoss physiology. Even though it will likely act as a rich source in functional genomics studies in years to come, surprisingly little is known about levels of genetic variability and structuring in this species. Here, we assess for the first time how genetic variation in *S*. *magellanicum* is spatially structured across its full distribution range (Northern Hemisphere and South America). The morphologically similar species *S*. *alaskense* was included for comparison. In total, 195 plants were genotyped at 15 microsatellite loci. Sequences from two plastid loci (*trn*G and *trn*L) were obtained from 30 samples. Our results show that *S*. *alaskense* and almost all plants of *S*. *magellanicum* in the northern Pacific area are diploids and share the same gene pool. Haploid plants occur in South America, Europe, eastern North America, western North America, and southern Asia, and five genetically differentiated groups with different distribution ranges were found. Our results indicate that *S*. *magellanicum* consists of several distinct genetic groups, seemingly with little or no gene flow among them. Noteworthy, the geographical separation of diploids and haploids is strikingly similar to patterns found within other haploid *Sphagnum* species spanning the Northern Hemisphere. Our results confirm a genetic division between the Beringian and the Atlantic that seems to be a general pattern in *Sphagnum* taxa. The pattern of strong genetic population structuring throughout the distribution range of morphologically similar plants need to be considered in future functional genomic studies of *S*. *magellanicum*.

## Introduction

Truly cosmopolitan species occurring at every continent and in all biomes are rare [1]. However, in some organism groups, such as birds and spore-producing plants, species have wide distribution ranges covering many, if not all continents and biomes [1,2]. Vicariance and/or long distance dispersal are the two main processes leading to wide, and often disjunct, distribution ranges. With advances in molecular methods, explanations involving the latter seems to be frequently supported, at least at the generic and specific levels [1,3–7].

Spore-producing organisms, such as lichens and bryophytes, have microscopic dispersal units, generally less than 40 μm [8,9]. Spores are usually wind-dispersed and they have the potential to colonise new habitats far from their origin [10]. Indeed, spore-producing organisms typically have wide distribution ranges [11–16] that span multiple continents [11,14], sometimes including both the Northern and Southern Hemisphere [6,17]. High genetic similarities among populations in widely separated regions are found in many bryophytes [18–21] and lichens [22,23]. Multiple founder events of remote islands also seem common in spore-producing plants [24,25], supporting the interpretation that long-distance dispersal occurs repeatedly.

Nevertheless, wide distribution ranges of morphologically-defined species do not necessarily reflect high dispersal abilities. Cryptic species occur within widely distributed spore-producing organisms [26–28]; phylogenetically distinct lineages are discovered without any obvious differences in morphology. As a result, some apparently wide-spread species could have more restricted ranges than previously assumed. Both bryophytes and lichens are structurally simple organisms often with few diagnostic morphological characters, and differentiating closely related species based on morphology alone can therefore be difficult. Genetic analyses sometimes indicate subdivision within species and careful re-examination of cryptic species might subsequently result in identification of morphological characters useful for distinguishing them [29,30]. Moreover, genetically differentiated groups or lineages found within species may occur in allopatry [31]. This indicates that there might be significantly more phylogenetic diversity than inferred from morphological variation in some groups of organisms.

*Sphagnum* is a nearly cosmopolitan genus, found on all continents except Antarctica. Many species in the genus have circumboreal distributions in the Northern Hemisphere, and a few occur disjunctively in the Southern Hemisphere. *Sphagnum magellanicum* Brid. (subgenus *Sphagnum*) is one of them, being one of the most globally widespread peatmosses. It is frequently used as model to understand peatmoss physiology [32], ecology [33], and phylogeography [19], and the genome of *S*. *magellanicum* is currently being sequenced and annotated (J. Shaw, D. Weston, unpublished). Hence, it will remain a model for ecological and evolutionary research, but also for genome-wide association studies (GWAS). Toward this end, the genetic structure of S. *magellanicum* at local, regional and global scales needs to be taken into consideration, not only for GWAS, but more generally, for knowing which taxon is being studied. To-date, the genetic architecture of this species across its global range is unknown.

*Sphagnum magellanicum* is the only species in subgenus *Sphagnum* with truly red gametophytes. Thus, there are few species that it can be confused with in field [34]. However, it can be difficult to separate from the somewhat reddish species *S*. *alaskense* R.E. Andrus & Janssens in areas where they co-occur [35,36]. *Sphagnum alaskense* was described from material collected along the western coast of North America a decade ago [35]. Later, it was found in eastern and northeastern Asia [36]. Gametophytes of *S*. *magellanicum* appear to be uniformly haploid [n = 19, 37], whereas chromosome number for *S*. *alaskense* is currently unknown. Plants of *S*. *alaskense* in Alaska were previously misidentified as *S*. *centrale* C. Jens [35], indicating that *S*. *alaskense* might be gametophytically diploid like *S*. *centrale* [37]. Both *S*. *magellanicum* and *S*. *alaskense* are dioecious; female and male gametangia (archegonia and antheridia, respectively) are separated on different gametophytes. Spore sizes ranges from 25 to 30 μm in *S*. *magellanicum* [38]. Sporophyte production is common, but likely varies between sites, regions, and years. In *S*. *alaskense*, sporophytes have not been reported [35], but have been observed in herbarium material from Alaska (herb. TRH).

Eastern North American and European populations of several widespread *Sphagnum* species, including *S*. *magellanicum* [19], are only weakly differentiated [19–21,39,40], probably because of ongoing gene flow across the Atlantic Ocean. A similar pattern of long-distance gene flow has been found for Asian and Alaskan plants [21,41]. However, continents seem to act as barriers in some circumboreal *Sphagnum* species and a fairly abrupt genetic break has been found in southeast Alaska, separating Alaskan specimens from conspecific plants to the south in western North America [21,41]. We hypothesise that similar genetic patterns occur in *S*. *magellanicum*, as it also has a circumboreal distribution range in the Northern Hemisphere. Thus, we predict genetic similarity between European and eastern American populations [19], similarity between Asian and Alaskan populations [41], but genetic differentiation between Atlantic versus Pacific plants, with a possible discontinuity in southeastern Alaska.

We aim to assess whether genetic variation in *S*. *magellanicum* is spatially structured across its range, and if so, to evaluate how historical factors and long-distance dispersal might have shaped observed patterns. We also include plants of the morphologically similar *S*. *alaskense* to determine whether these two morphologically similar species are separated genetically and if they have different ploidy levels.

## Materials and Methods

*Sphagnum magellanicum* is common and often the dominant peatmoss in ombrotrophic mires in the southern arctic, boreal, and nemoral bioclimatic zones in the Northern Hemisphere. In the Southern Hemisphere, *S*. *magellanicum* occurs throughout South America. At higher elevations it occurs in tropical alpine [42] and cloudy subalpine areas [43,44], while in the southern parts of Argentina and Chile, *S*. *magellanicum* mainly occurs in the northern antiboreal bioclimatic zone [45]. The large ombrotrophic mires in Tierra del Fuego are often totally dominated by *S*. *magellanicum* [46], likely due to the absence of competition from other sphagna as it is often the only peatmoss present. In fact, the species was described from material collected at Cape Horn [47]. The subspecies *S*. *magellanicum* subsp. *grandirete* (Warnst.) A.Eddy has been reported from Madagascar [48], but its taxonomic status is unclear.

The main habitat of *S*. *magellanicum* in the Northern Hemisphere is bog (ombrotrophic) and poor fen (minerotrophic) mire communities, and it is mostly absent from rich fens [38,49]. It occupies a wide range along the ‘dry-wet’ mire ecogradient, as it grows in low hummocks, lawns and carpets [49]. In ombrotrophic mires of Tierra del Fuego, it occupies all habitats along the ‘dry-wet’ ecogradient from the driest hummocks to the wettest carpets and pools [46]. *Sphagnum magellanicum* also occurs in moist heaths, on mineral soil in forests and on rock walls in oceanic regions of the Northern Hemisphere. In cloudy high altitude subalpine forests of Costa Rica it can form extensive carpets and small hummocks in small mires on shallow peat, but occurs more commonly around the margins of moraine lakes [43,44]. In the northern Andes, it occurs partly in nutrient poor mires with underlying peat, but it also grows directly on the bedrock or on non-organic soils, with little or no peat accumulation, and sometimes as extensive carpets on vertical cliff faces [50,51].

*Sphagnum alaskense* is found growing in poor to medium fens and mineral edges of ombrotrophic mires in western North America [34]. The habitat of *S*. *alaskense* from western Asia [36] is more obscure (reported from bogs, lake shores, and boggy forests), because of ambiguities in mire terminology. Nearly all collections of *S*. *alaskense* from western North America in herb. TRH (10 specimens) are from poor and medium rich mire hummocks and lawns of mire margins, and a few collections are from hummocks in forested peatland. It seemingly avoids ombrotrophic (bog) mire sites. This is contrary to haploid *S*. *magellanicum*, which is a member of both ombrotrophic and minerotrophic mires, and grows in open mire expanses as well as along mire margins.

### Sampling Strategy

We sampled plants of *S*. *magellanicum* from herbarium collections to cover as much of its geographical distribution as possible, and most of the species’ habitat range and morphological variation were covered as well. Broad sampling both spatially and ecologically increases the chance of finding genetically divergent lineages within *S*. *magellanicum* [52]. Four herbaria were visited for collection: DUKE (Durham, USA), LE (St. Petersburg, Russia), MHA (Moscow, Russia), and TRH (Trondheim, Norway). Additionally, a few samples were obtained from herbaria MA (Madrid, Spain) and BING (New York, USA). Altogether, 220 collections labelled *S*. *magellanicum* and 25 collections labelled *S*. *alaskense* from western North America were sampled. All samples collected were verified morphologically. From each collection, one shoot was picked for DNA analyses.

### Molecular Analyses

A small piece from the central part of the shoot apex was used for DNA extraction. Extractions were performed using either the CTAB protocol described in Shaw *et al*. [53] or DNeasy 96 Plant Kit (Qiagen, Oslo, Norway) following the manufacture’s protocol (except in the last step where 50 μL, instead of 100 μL, elution buffer was added twice).

Fifteen microsatellite markers developed for *Sphagnum* were amplified in *S*. *magellanicum*. Microsatellite names and primers are provided in Shaw *et al*. [54] and Stenøien *et al*. [20]. Three to four markers were amplified in 8 μl multiplex reactions using Qiagen Multiplex PCR Kit (Qiagen, Oslo, Norway). The loci used were marked with fluorophores (HEX, FAM and NED) and divided in four mixes according to expected length, as follows: mix 1: loci 1, 7, 12, 68; mix 2: loci 4, 10, 30; mix 3: loci 19, 22, 29, 93; mix 4: loci 9, 14, 20, 56. The thermocycling regime started with an initial step at 95°C for 15 minutes, followed by 33 cycles at 94°C for 30 seconds, 53°C for 90 seconds, and 72°C for 60 seconds, and finished with a final step at 60°C for 30 minutes. 1 μL of PCR product, 8.85 μL of Hi-Di^™^ Formamide (Applied Biosystems, Norway) and 0.15 μL GSLizz500 were mixed for electrophoresis on an ABI 3730 sequencer. GENEMAPPER^®^ software (Applied Biosystems) was used to genotype the alleles.

Two loci from the plastid genome, *trn*L (UAA) 59 exon-*trn*F (GAA) and tRNA(Gly) (UCC), hereafter *trn*L and *trn*G, respectively, were sequenced from a subset of samples from the microsatellite dataset. Thirty-two samples were chosen for DNA sequencing based on microsatellite variation (see below) and geographical distance. PCR amplifications were carried out using puReTaq Ready-To-Go PCR Beads (Amersham Biosciences) in solutions of 22.8 μL H_2_O, 0.1 μL forward primer, 0.1 μL reverse primer, and 2.0 μL DNA extract. The PCR cycle profile was as follows: 95°C for 5 minutes, 51°C seconds for 45 seconds, 72°C for 45 seconds, with step 2 and 3 repeated 35 times, 72°C for 5 minutes. For *trn*L, step 2 and 3 were as follows: 54°C for 45 seconds, 72°C for 190 seconds.

### Statistical Analyses of Microsatellite Data

Population structure was explored using clustering analyses implemented in Structure 2.3.4 [55–58]. A Bayesian approach is used in Structure to identify genetically homogeneous groups of specimens. The analysis was performed using 50,000 iterations as burn-in followed by 200,000 iterations. This was replicated ten times for a set of genetic clusters (*K*) with a maximum of 10. The Structure results were analysed, summarised, and visualised using the online version of Clumpak [59]. The best *K* was also estimated by the Clumpak option “Best *K*”. This method uses the likelihood values of all *K* values to identify the most likely number of clusters in the dataset. The results of the Structure analyses were plotted on maps using the R packages maps and plotrix in the R Environment [60]. Genetic structure was further explored by principal coordinate analyses (PCA) using GenAlEx 6.501 [61,62].

Genetic variation and distance measures were estimated for the data in two ways: (1) samples grouped by geographical origin and (2) genetically-based groups inferred from cluster analyses. For all geographical and genetic groups the percentage of polymorphic loci, expected heterozygosity, and mean number of alleles were estimated, and pairwise *F*_ST_ and Nei’s genetic distances between the groups were calculated using GenAlEx [61,62].

### Phylogenetic Relationships

Nucleotide sequences from two plastid loci were used to reconstruct the phylogenetic relationships among samples of *S*. *magellanicum* and *S*. *alaskense*. All sequences were aligned using ClustalW with default parameters in Mega 6.0 [63]. Insertions were coded as characters according to Simmons and Ochoterena [64]. Phylogenetic relationships were reconstructed using the Maximum Likelihood option in Mega 6.0, adding 1000 bootstrap replications and the general time reversible substitution model (GTR; the same results were obtained using Jukes-Cantor model). In addition, a haplotype network based on the sequences were reconstructed to show number of mutational steps between haplotypes obtained, using the software TCS [65].

### Divergence Time Estimation

An isolation-with-migration model was used to estimate population divergence time (*T* = *t*μ, where *t* is divergence time in years and μ is mutation rate per year) between the “orange” and “blue” genetic groups inferred by Structure (see below) using IMa [66]. These two groups have overlapping distributions and are represented by many individuals. Both microsatellite markers (number of repeats at each locus) and *trn*L sequences were included in the analysis. A preliminary test was performed following the recommendations in the user manual, while the full scale analysis was performed using 100,000 steps as burn-in followed by 20 mill steps. A geometric heating scheme with parameters set to 0.8 and 0.9 and 30 Metropolis-coupled chains was applied. The upper boundary for population sizes were set to 0.5 and divergence time to maximum 5. The migration parameters were excluded to increase statistical power.

## Results

One hundred-ninety-five samples were successfully amplified for 14 microsatellite loci (see S1 File for list of voucher specimens). Samples from the remaining herbarium specimens failed to amplify likely due to degraded DNA, and microsatellite marker 9 was excluded as 1/3 of the samples had missing data (S2 File). Fifty-nine of the *S*. *magellanicum* plants were diploid based on the observation that 50% or more of the microsatellite loci had two alleles [67]. Similarly, all *S*. *alaskense* plants (n = 22) were diploid, as two alleles were found for each sample in 10 of 14 microsatellite loci. Only two loci were fixed for one allele among all diploid samples.

All samples of *S*. *magellanicum* and *S*. *alaskense* were analysed together using the software Structure. With *K* = 3, the diploid formed one distinct genetic group, while haploid data were divided in two other groups (results not shown). One Chinese individual with only three heterozygous loci grouped with diploid samples at all *K* values in the Structure analysis. Thus, this sample was considered to be diploid, but with missing alleles. Four diploid samples grouped together with haploid genetic groups. However, these individuals were heterozygous in more than 50% of the loci and, thus, interpreted as diploids. The data were divided in two datasets (one haploid and one diploid), and further analysed separately. One hundred-eleven haploid plants and 82 diploid plants were analysed, respectively.

### Haploid *S*. *magellanicum*

**Fig 1:**
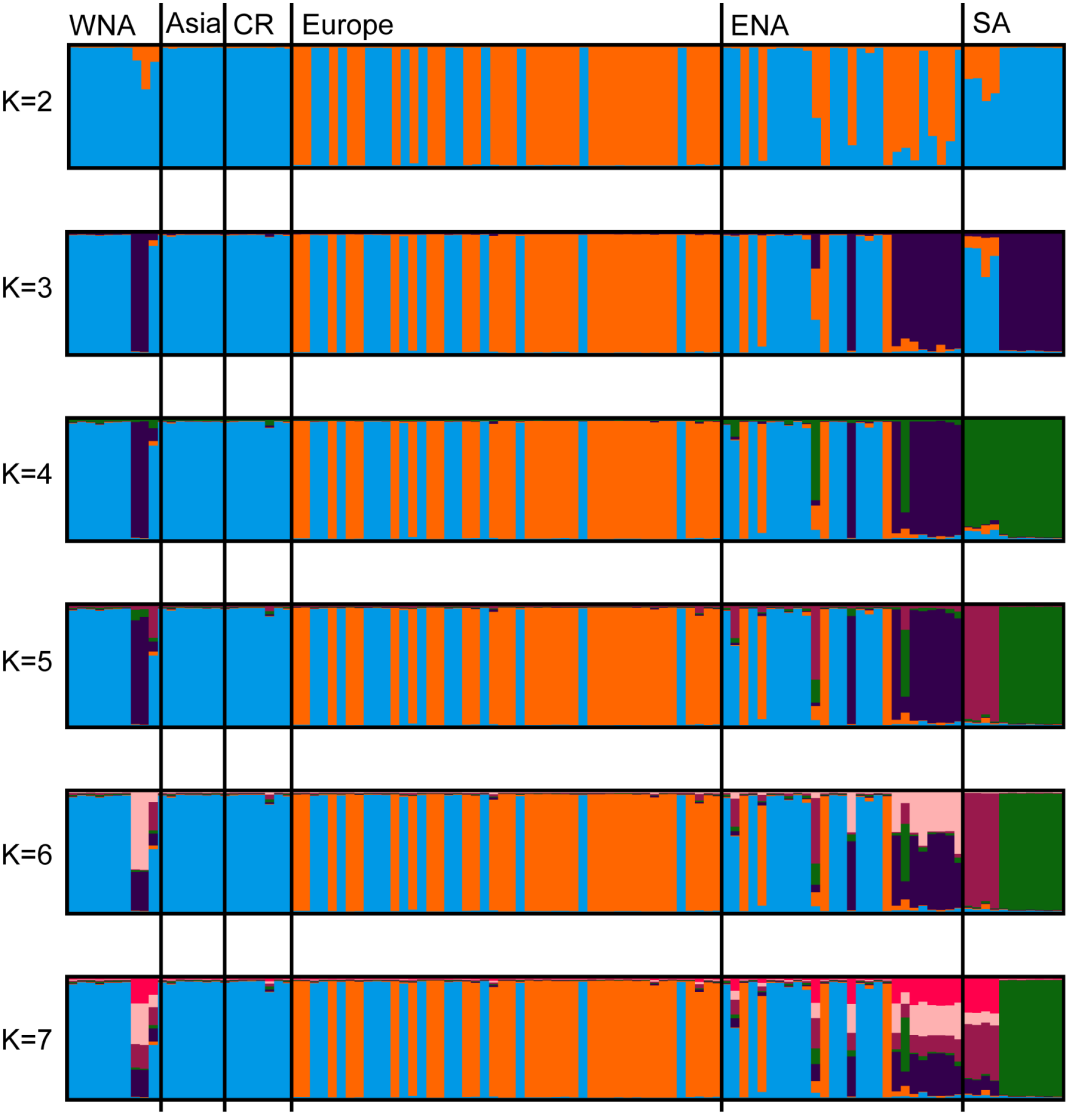
Structure results comparing *K* = 2 to *K* = 7 for haploid samples of *Sphagnum magellanicum*. The number of genetic clusters (*K*) are given to the left of the barplots, while the regions the samples are collected in are above the first barplot and divided by black lines. Abbreviations: WNA-western North America, CR-Central Russia, ENA-eastern North America, SA-South America.

**Fig 2:**
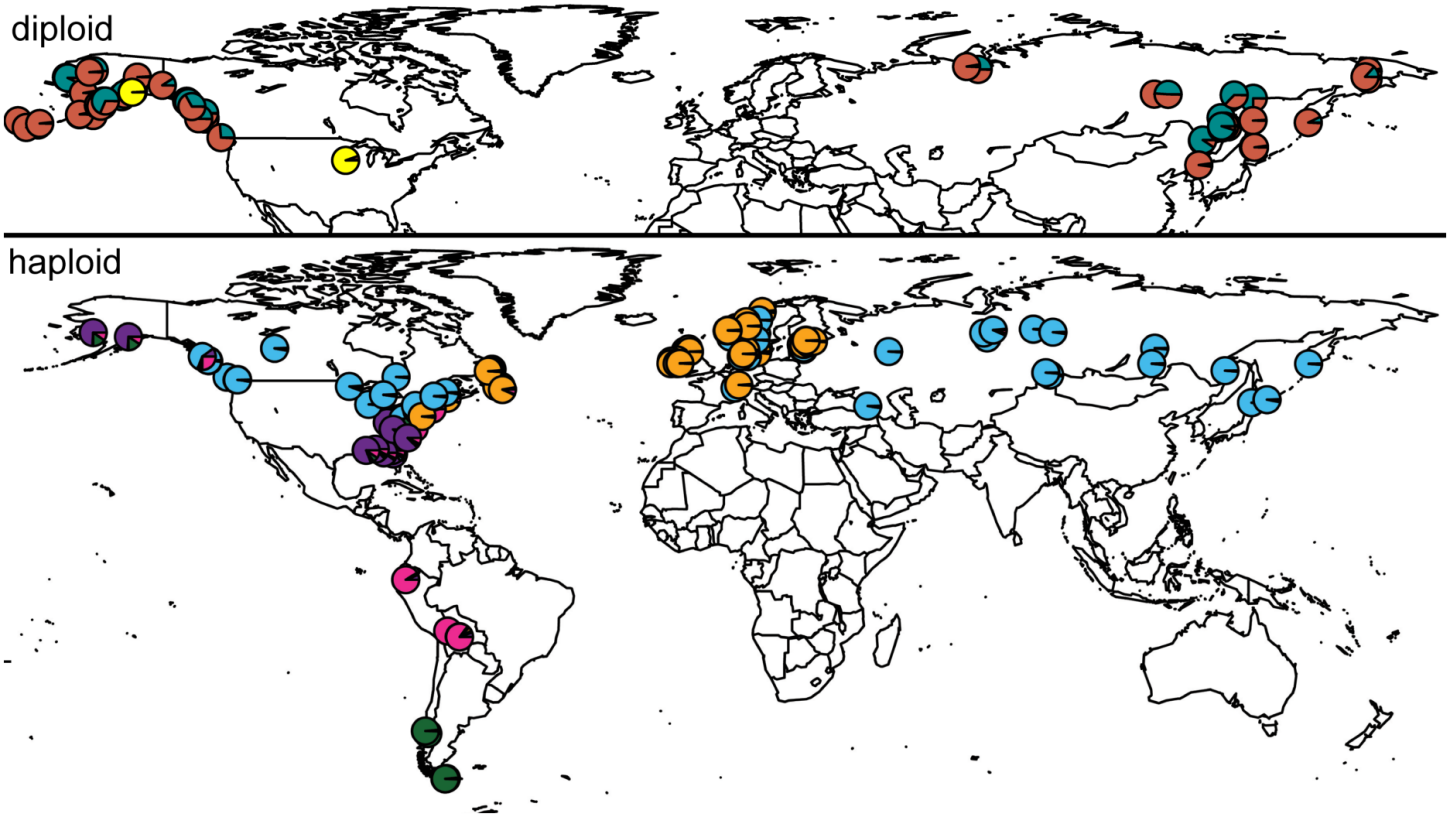
Geographical distribution of genetic groups inferred by the software Structure for all samples of haploid *Sphagnum magellanicum* (below, colours as in Fig 1) and all samples of diploid *S*. *magellanicum* and *S*. *alaskense* (above). Genetic groups in the haploid plants differ in their total geographical distributions, but no spatial structure was found for diploid plants.

Genetic structure among haploid *S*. *magellanicum* plants was inferred using Structure (Figs 1 and 2). The most likely number of genetic groups estimated by Best *K* in Clumpak was *K* = 5 (Prob(*K* = 5) = 0.99). The probability of *K* = 6 was 0.01, while the probability of all other *K* values was 0. A comparison of Structure results for *K* = 2–7 is shown in Fig 1. Using *K* = 5 (Fig 2), the South American samples include two genetic groups, one southern (“green” cluster) and one northern (“pink” cluster). Three genetic groups occur across the Northern Hemisphere. Most plants belong to one of two widespread Northern Hemisphere groups, “orange” and “blue”. The “orange” group occurs only in the Atlantic region, whereas the “blue” group is spread across the Northern Hemisphere. Most of the plants collected in the southeastern United States plus two samples from Alaska form a distinct genetic group (“purple”). Three individuals from eastern North America (Virginia, Connecticut, and Newfoundland) are admixed with South American clusters.

**Fig 3:**
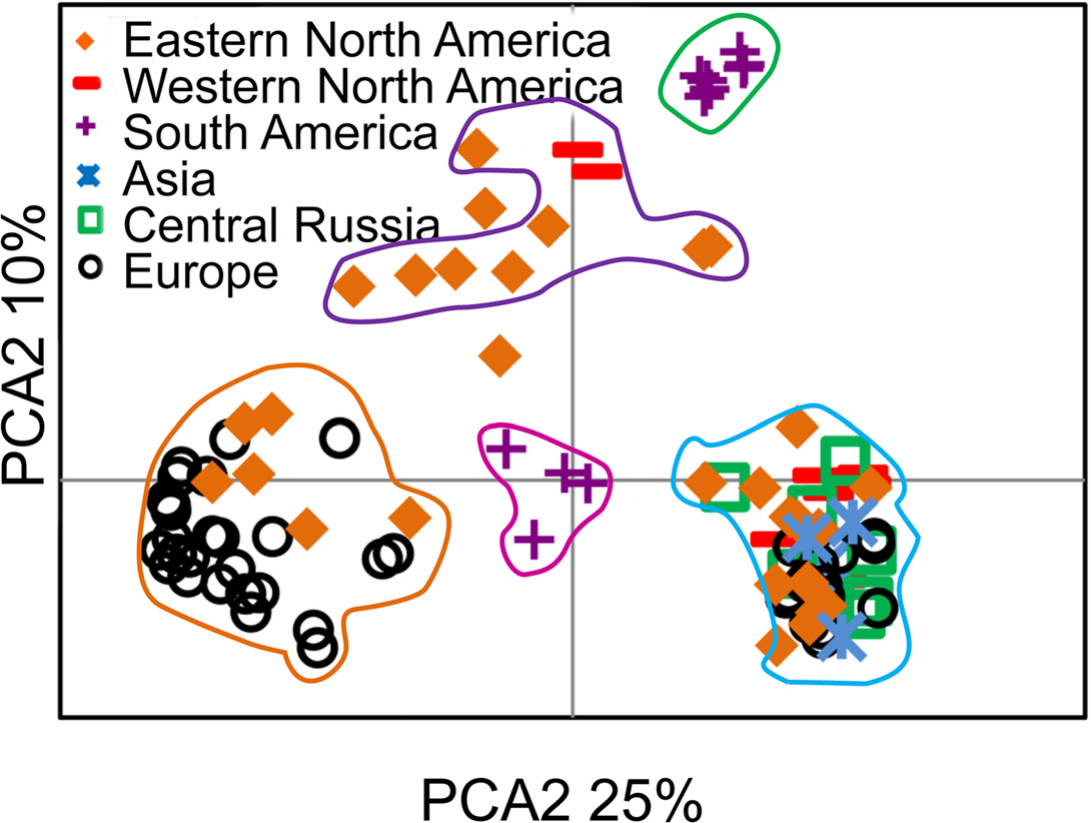
Principal coordinate analysis based on microsatellite loci of six groups of haploid *Sphagnum magellanicum* divided in geographical regions. Coloured symbols in the upper left corner show geographical origin of the samples and the coloured lines correspond to different genetic groups inferred by Structure (same colours as used in Fig 2 lower map). The dots that are not enclosed are admixed between different genetic groups.

The principal coordinate analysis is shown in Fig 3. The results correspond to the Structure results. Two main groups were detected, one containing amphi-Atlantic specimens and another with samples located throughout the Northern hemisphere. All individuals within the same genetic cluster (*K* = 5) inferred by Structure, group together in the PCA plot (indicated by colours in Fig 3).

Genetic diversity measurements were estimated excluding microsatellite marker 4, as this marker did not amplify in one of the genetic groups. This marker is fixed for one allele so no evolutionary signal was lost. Genetic diversity is highest in eastern North America and lowest in Central Russia and Asia (Table 1). Several genetic groups are represented in the eastern part of North America, while only one group is found in Asia. All samples from South America were pooled together in one regional population, resulting in relatively high genetic diversity in this region. However, the “green” and “pink” group show low genetic diversity (Table 2). The “purple” group is twice as variable as the “blue” and “orange” groups (Table 2). Two samples from Alaska were included in the “purple” group based on microsatellites, but differ from the other “purple” individuals in plastid DNA markers (see below). Estimates excluding these two samples from the “purple” group, did not affect inferences about genetic diversity (results not shown). The “green” cluster is the least variable group.

**Table 1:** Genetic diversity indices for haploid *S*. *magellanicum*.

Region	N	N_A_	*H*_E_	PPL %
Western North America	9	2.69±0.36	0.42±0.06	92
Asia	5	1.77±0.28	0.24±0.08	46
Central Russia	9	1.85±0.34	0.20±0.08	38
Europe	45	3.62±0.71	0.47±0.07	85
Eastern North America	29	5.69±0.97	0.64±0.06	92
South America	11	2.77±0.48	0.43±0.06	92
Total	108	3.06±0.27	0.40±0.03	74+10

Number of samples (N), mean number of alleles (N_A_), expected heterozygosity (*H*_E_), and proportion of polymorphic loci (PPL %) for haploid *S*. *magellanicum* divided in geographic groups. Samples with more than 40% missing data were excluded from the analysis.

**Table 2:** Genetic diversity indices for genetic groups inferred by Structure in haploid *S*. *magellanicum*.

Group	N	N_A_	*H*_E_	PPL %
Blue	47	4.00±1.19	0.32±0.10	54
Orange	37	3.10±0.67	0.32±0.08	69
Purple	10	3.85±0.50	0.60±0.06	92
Pink	4	1.62±0.18	0.25±0.07	54
Green	7	1.62±0.37	0.15±0.08	23
Total	105	2.83±0.32	0.33±0.04	58±11

Number of samples (N), mean number of alleles (N_A_), expected heterozygosity (*H*_E_), and proportion of polymorphic loci (PPL %) for haploid *S*. *magellanicum* divided in genetic groups inferred by the software Structure. Three admixed individuals were excluded from the analysis.

South America seems to be less differentiated from North American regions, than from Eurasian regions. Between the Northern Hemisphere regions, the *F*_ST_ values are relatively low, except between Europe and other regions (see S3 File for results). Genetic distance estimations between genetic groups are shown in Table 3. All pairs of genetic groups are strongly differentiated as shown by both high Nei’s genetic distances and *F*_ST_ values.

**Table 3:** Nei’s genetic distance (below diagonal) and *F*_ST_ (above diagonal, significant values in bold) for pairs of genetic groups inferred by the software Structure for haploid *S*. *magellanicum*.

Group	Blue	Orange	Purple	Pink	Green
Blue		**0.50**	**0.42**	**0.42**	**0.51**
Orange	0.88		**0.40**	**0.42**	**0.61**
Purple	0.97	1.03		**0.33**	**0.40**
Pink	0.61	0.75	1.48		**0.65**
Green	0.73	1.58	0.95	1.23	

Three admixed individuals were excluded from the analysis.

### Diploid *S*. *magellanicum* and *S*. *alaskense*

**Fig 4:**
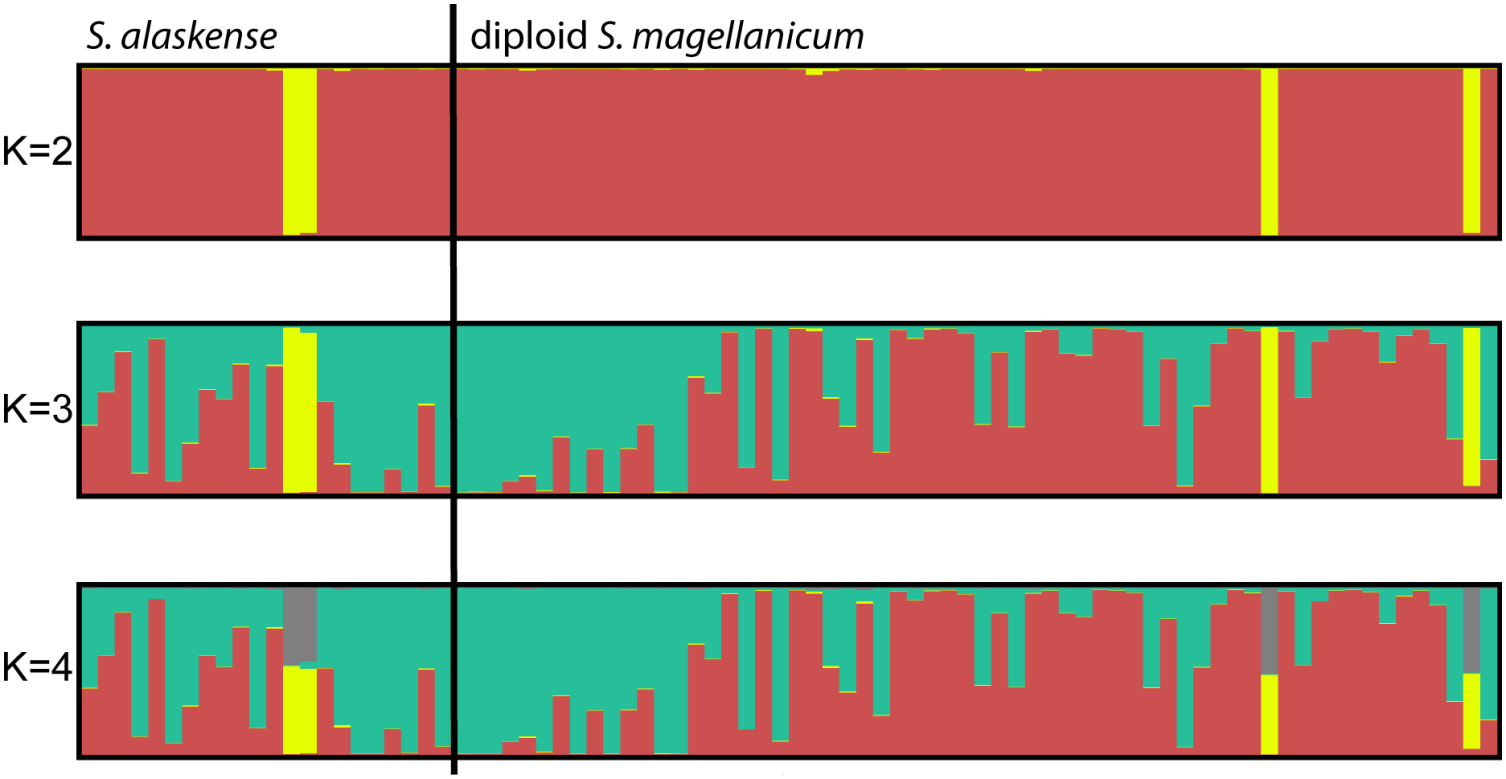
Structure results comparing *K* = 2 to *K* = 4 for diploid samples of diploid *Sphagnum magellanicum* and *S*. *alaskense* (colours as in Fig 2). The number of genetic clusters (*K*) are given to the left of the barplots.

All diploid plants are restricted to western North America and Asia, with two outliers in Central Russia and one in Iowa, USA. The diploid *S*. *magellanicum* samples co-occur with *S*. *alaskense* in western North America. The Best *K* estimation showed that there are likely three genetic groups (Prob(*K* = 3) = 0.99) across all diploid samples. Four samples form a separate group (“yellow”, Fig 4). These samples grouped with haploid plants when the full dataset was analysed (see above). We found no clear separation between plants identified as *S*. *alaskense* versus diploid *S*. *magellanicum*, but many samples belong to either a “red” or a “turquois” genetic group. No geographical structure was found (Fig 2A). The principal coordinate analysis revealed a closely comparable pattern (results not shown).

Genetic diversity is similar in *S*. *alaskense* and diploid *S*. *magellanicum*, *H*_E_ = 0.53 (±0.07) and 0.50 (±0.08), respectively. The mean number of alleles per locus (*N*_A_) is 5.14 (±0.08) in *S*. *alaskense* and 6.14 (±1.32) in diploid *S*. *magellanicum*. The percentages of polymorphic loci are the same (86%). Nei’s genetic distance between the two is 0.02 and *F*_ST_ was 0.01.

### Phylogenetic Relationships

**Fig 5:**
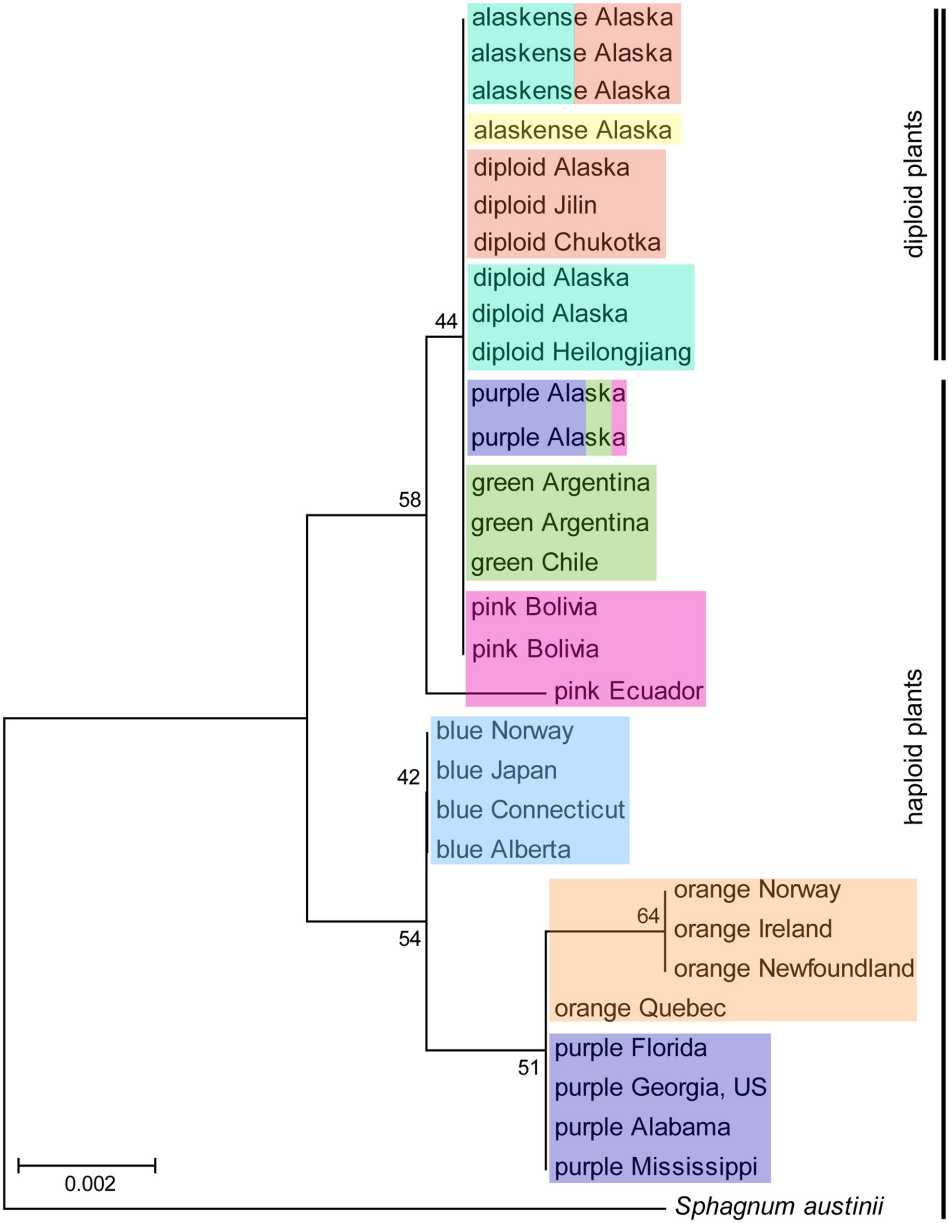
Maximum likelihood tree based on chloroplast DNA marker *trn*L for a subset of *Sphagnum magellanicum* (both haploids and diploids) and *S*. *alaskense* samples representing all genetic groups inferred by the software Structure. The different genetic groups are indicated with colours corresponding to the ones used in Fig 2 (both maps). Another species from the subgenus *Sphagnum*, *S*. *austinii*, was used to root the tree.

**Fig 6:**
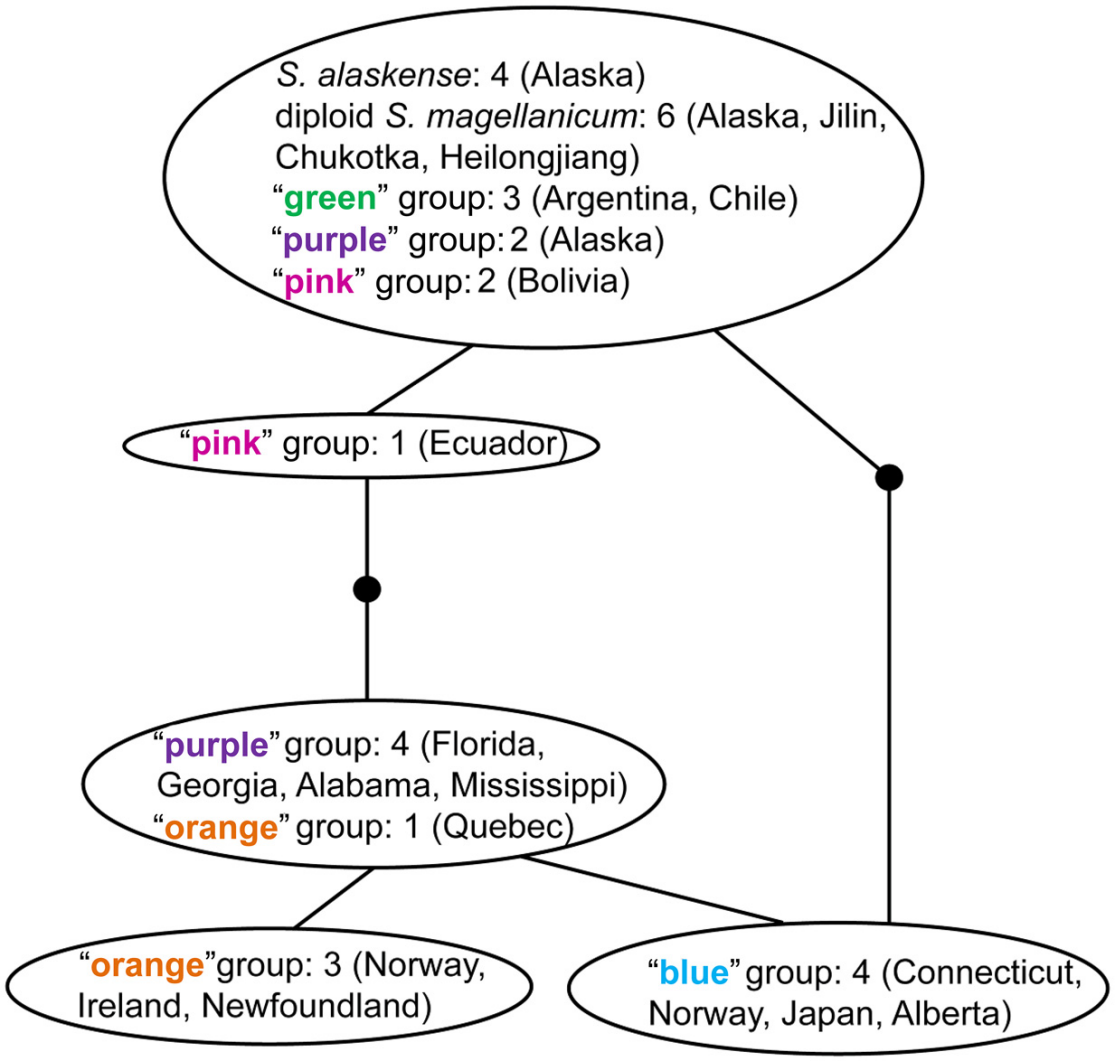
Genealogical relationships based on chloroplast DNA marker *trn*L for a subset of *S*. *magellanicum* and *S*. *alaskense* samples. The size of the ovals are proportional to haplotype frequencies. The number of plants are given in each oval for each genetic groups (inferred by Structure based on microsatellites) of haploid plants and for diploid plants (*S*. *alaskense* and diploid *S*. *magellanicum*). Lines and dots indicate one mutational change, and dots represent unsampled haplotypes.

All but three specimens share the same haplotype at the *trn*G locus. Two “blue” haploid specimens differ from this haplotype by one substitution and one “purple” haploid plant differ by another substitution. Thus, *trn*G was not included in phylogenetic analyses. For *trn*L, five haplotypes (separated in total by two insertions and three substitutions) were found (Figs 5 and 6). All diploid plants, including *S*. *alaskense*, the two haploid plants from Alaska (“purple”), and South American plants share two insertions in their sequences and are identical, except for a plant from Ecuador that differs in one substitution. All haploid samples in the Northern Hemisphere form a clade (no insertions). The three genetic groups, “blue”, “orange”, and “purple”, have different haplotypes, except one “orange” plant sharing the “purple” haplotype. The haplotype network (Fig 6) shows the number of mutational changes between all *trn*L haplotypes. Nucleotide sequences are available in GenBank (see S4 File for accession numbers).

### Divergence Time Estimation

Divergence time between the “orange” and the “blue” haploid genetic groups was estimated as 0.28 (95% CI = 0.11–1.49). Using a mutation rate of 4.4x10^-6^ estimated for microsatellite markers in *Sphagnum* [68] and a mean mutation rate of 5x10^-4^ per site per Mya for chloroplast nucleotides used in other molecular dating studies in mosses [69], converted to mutation rate per gene per year, the divergence time between the groups in years was found to be approximately 76,400 years BP (95% CI = 29,000–403,600).

## Discussion

Despite their apparently overall lack of worldwide morphological differentiation, many widely distributed Northern Hemisphere peatmosses are divided genetically into Atlantic and Beringian groups [21,41]. We found the same pattern for *S*. *magellanicum*. Surprisingly, in this species the pattern is revealed at the ploidy level; diploid plants belong to the Beringian group whereas haploid plants form a broad Atlantic group. Haploid plants of *S*. *magellanicum* are further divided in five genetic groups based on microsatellite makers and these groups differ in distribution ranges. Our findings indicate that gene flow in the widely distributed *S*. *magellanicum* is limited between the various genetic groups, and little admixture is evident.

*Sphagnum magellanicum* could potentially include several individual species based on our findings [70]. Genetic differentiation are high between genetic groups of haploid *S*. *magellanicum* compared to other *Sphagnum* species with comparable distribution ranges and genetic diversity levels [21]. However, as we have only looked at genetic data, we use the term genetic groups about potential new taxa when discussing our findings. To further evaluate the taxonomical status of the genetic groups, careful morphological examination should be applied to determine if genetic groups are cryptic or not.

### Distribution Ranges of Genetic Groups

The five genetic groups inferred among haploid *S*. *magellanicum* samples have different geographical ranges. Only the “blue” and “orange” groups overlap and their distribution patterns resemble those of the two closely related species *S*. *beothuk* and *S*. *fuscum*, with the former restricted to the Amphi-Atlantic region, whereas the latter is found across the Northern Hemisphere [71]. Only four other amphi-Atlantic *Sphagnum* species are known: *S*. *affine* [40], *S*. *angermanicum* [20], *S*. *beothuk* [71], and *S*. *venustum* [38,72]. The “orange” group within *S*. *magellanicum* could potentially be another amphi-Atlantic *Sphagnum* species. In Norway, the “orange” group seems to mainly occupy mire expanse sites, whereas the “blue” group usually is found along mire margins. However, based on field observations, the “blue” group probably has a wider habitat range than the “orange”, at least in areas where the latter is absent.

The distribution of the “purple” group is disjunct, with two specimens in Alaska, but the majority of plants occur in southeastern North America. Other *Sphagnum* species also have their main distributions in southeastern North America, for example *S*. *fitzgeraldii* and *S*. *cyclophyllum* [73]. However, *S*. *fitzgeraldii* has a disjunct occurrence in Galapagos Islands, South America, and *S*. *cyclophyllum* is found further north along the eastern coast of North America than the “purple” *S*. *magellanicum*. The two South American groups within *S*. *magellanicum* are geographically allopatric, with the “green” group confined to the southernmost parts and the “pink” occurring in the northern parts. Similarly, the widespread lichen *Cetraria acuelata* forms one southern and one northern genetic group in South America [6]. We have few samples from South America; thus, more data are needed to confirm whether genetic structuring observed in South America is a consistent pattern in *S*. *magellanicum*.

All but three plants of *S*. *magellanicum* sampled from Alaska are diploid. Additionally, the majority of plants we examined from eastern Asia are diploid, suggesting that the haploid *S*. *magellanicum* probably is rare in the northern Pacific region. This supports the view of Maksimov and Ignatova [36] who reclassified all *S*. *magellanicum* plants in northeastern Asia as *S*. *alaskense*. One diploid plant collected in Iowa, U.S.A, together with two samples from southern Yamal Peninsula, Russia, are outliers in the otherwise amphi-Pacific distribution of the diploid plants.

### Historical Factors and Long-Distance Dispersal

The last glacial maximum influenced current species distributions and genetic diversity patterns in the Northern Hemisphere [74]. The “orange” and “blue” genetic groups in *S*. *magellanicum* appear to have split before the last glacial maximum. As most genetic groups are differentiated at approximately the same level as the “blue” and “orange” genetic groups as shown by high *F*_ST_ values, the genetic groups may have differentiated because of separation in different glacial refugia with no gene flow among them. The genetic groups differ in their distribution ranges, thus, they may have had different abilities to disperse and colonise after the last glacial maximum. This could be due to differences in spore production, limitations to spore dispersal by for example wind currents, or limitations to the establishment of spores [75].

Despite being a major refugium for many plants [76], it appears that few haploid *S*. *magellanicum* survived the last glaciation in Beringia, as seen by their rarity in the region today. On the other hand, the present distribution of diploid plants indicates glacial survival in Beringia or eastern Asia with Holocene expansion into most of the Pacific region. The haploid “purple” group is currently found in an area that remained ice-free for the entire glacial period. The distinct alleles and high genetic diversity in this group indicate that it may well have survived in southeastern parts of North America. Also, survival in eastern North American refugia is likely for the “orange” group, with post-glacial colonisation of Europe across the Atlantic Ocean [40]. Alternatively, the “orange” group survived in Europe and later colonised the east coast of North America [19]. Both the “orange” and the “blue” groups have northern distributions compared to the genetically more variable southern “purple” group, suggesting that the former groups were more affected by the glaciation, possibly including population bottlenecks.

Allodiploid *Sphagnum* species usually have higher levels of genetic diversity than haploid species because of the fixation of two alleles at many loci, see for example [77]. It is therefore somewhat surprising that the “purple” haploid group of *S*. *magellanicum* is more diverse than any of the diploid groups. Allopolyploids are not as sensitive to reduction of genetic variation following bottlenecks because of fixed heterozygosity [78]. Thus, relatively low levels of genetic diversity in diploid *S*. *magellanicum*/*S*. *alaskense* compared to other allodiploid *Sphagnum* species could be caused by hybrid origin of few individuals of closely related species.

Both genetic groups found in South America have low levels of genetic diversity. Low genetic variation may be a consequence of recent establishment of one or few haplotypes following long-distance dispersal from the Northern Hemisphere [79]. However, low genetic variation might also have been caused by limited sampling (n = 11). On the other hand, plants sampled from sites more than 1000 kilometres apart are genetically quite uniform. All plants share the same plastid haplotype, which is identical to the haplotype found in the diploid plants of *S*. *magellanicum*, indicating that the establishment in South America happened relatively recently [17]. Dispersal of plants from the Northern to the Southern Hemisphere has been hypothesised to happen either stepwise along the Andean mountain range or by migratory birds [3,80]. A *Sphagnum* fragment has recently been found in the plumage of a bird migrating between the Northern and Southern Hemispheres [81], indicating that this could be a dispersal vector for bryophytes across the equator. Indeed, it has been suggested that this is how plants of the moss genus *Tetraplodon* reached South America [4].

### Phylogenetic Relationships

Species within the genus *Sphagnum* are relatively young. Even though the clade is old, species diversification likely took place in the Northern Hemisphere during climate cooling in the late Tertiary [82]. We found little differentiation in plastid DNA within *S*. *magellanicum* comparing different genetic groups defined by microsatellite data. However, even though the genetic differences found may seem small, together with nDNA differentiation they may nonetheless indicate ongoing or recent speciation in this widespread species [70].

The Northern Hemisphere haploid groups constitute one clade, while plants from South America share the exact same plastid sequence as diploid, except one specimen from Ecuador that differs with one substitution. The two “purple” individuals sampled from Alaska might not be as related to the plants from the southeastern United States as inferred from Structure based on microsatellites. Rather, they share plastid DNA with the diploid and South American plants. Plants from southeastern North America assigned to the “purple” group seem to be closely related to plants in the “orange” group based on plastid DNA. The distributions of these two groups overlap slightly in eastern North America. The sharing of one plastid DNA haplotype could indicate recent speciation, with too little time for complete linage sorting [83]. The liverwort *Frullania asagrayana* is also divided in southern and northern groups in eastern North America based on microsatellites, but they do not differ in nucleotide sequences [27]. The divergence of the two *F*. *asagrayana* groups was hypothesised to be associated with the Pleistocene glaciations. This could also be the case for the Northern Hemisphere genetic groups we resolved within *S*. *magellanicum*; separation in different refugia with no gene flow during the last glaciation and secondary contact and/or overlapping distributions in the Holocene following post-glacial colonisation.

### Origin of Diploid *S*. *magellanicum* and *S*. *alaskense*

We were not able to find any distinction between the diploid plants named *S*. *magellanicum* and those named *S*. *alaskense* using microsatellite or plastid DNA markers. The fact that most plants of *S*. *magellanicum* from Alaska and northeast Asia are diploid and genetically similar to *S*. *alaskense* likely explains why the two can be difficult to separate in field, and indicate that they may belong to the same taxon. Preliminary morphological examinations indicate that *S*. *alaskense* plants seem to differ somewhat from diploid *S*. *magellanicum*. This is most easily seen by the more slenderly pointed branches in the outer part of the capitula of the former than the latter. *Sphagnum alaskense* also seems to have more imbricate branch leaves. However, this differentiation is not correlated with genetic patterns in any of the markers used here. Morphological differences with no genetic differentiation was similarly found within *S*. *palustre* L. [25] and phenotypic plasticity was hypothesised to underlie the different morphs.

Other allodiploid *Sphagnum* species have been confirmed using microsatellite markers; for example, *S*. *troendelagicum* [77], as they often are fixed for two alleles at each locus, one inherited from each parental species. Combining diploid and haploid *S*. *magellanicum* and *S*. *alaskense* in Structure analyses did not resolve any potential parents among the haploid genetic groups. Haploid *S*. *lescurii* and the allodiploid *S*. *missouricum* also formed different genetic groups based on microsatellites [84] even though haploid *S*. *lescurii* is the maternal parent of the diploid plants [85]. Two diploid *S*. *magellanicum* plants from Iowa and Alaska, U.S.A, and two plants of *S*. *alaskense* from British Columbia, Canada, were admixed between haploid genetic groups. Morphological examination shows that the Iowa sample is somewhat different from other diploid *S*. *magellanicum*, but still falls within that morphological group. These four samples might reflect independent hybridisation events.

To further evaluate if the diploid *S*. *magellanicum* and *S*. *alaskense* are conspecific or indeed different taxa, a thorough comparison of morphological characters has to be done together with molecular analyses using other molecular markers. Until then, all diploid plants of *S*. *magellanicum* should be considered to belong to *S*. *alaskense*.

## Conclusion

Our results provide further evidence that widely distributed peatmosses are genetically structured across their distribution ranges [21,41]. The processes acting on shaping the separation of the “Beringian” and “Atlantic” groups may also shape similar genetic patterns in other *Sphagnum* species or even in spore-producing organisms in general. The wide distribution ranges of some *Sphagnum* species may be more limited than previously assumed based on morphological uniformity. Rather than circum-boreal distributions in *Sphagnum*, there seem to be main two ranges characterising genetic groups within morphospecies: one covering Asia and Alaska (except the southernmost part) and one mainly occurring in the Atlantic region, but with extensions into western North America (from southern Alaska and southwards) and through Russia into southeastern Asia.

Whether genetic groups of *S*. *magellanicum* represent cryptic species, or merit formal taxonomic recognition at specific and/or infraspecific rank, requires examination of morphological characteristics that can be used to separate them. Especially, clarifying the status of the “orange” and “blue” genetic haploid groups is important as the groups overlap in the Atlantic region. Pooling them together in for example ecological or genomic studies could give misleading results if they indeed belong to different taxa. Our results show that widespread *Sphagnum* species may represent lack of morphological divergence and possibly cryptic speciation, rather than being the result of ongoing long-distance dispersal.

## Supporting Information

S1 File List of voucher specimens of *S*. *magellanicum* and *S*. *alaskense*. (PDF)

S2 File Number of herbarium collections sampled (Collections), number of samples included in genetic analyses (Haploid), number of diploid specimens detected in molecular analyses (Diploid), number of misidentified samples (Misidentified) confirmed based on both genetic data and morphological examination, and number of samples that did not amplify (No DNA) of *Sphagnum magellanicum* and *S*. *alaskense* (all collections from Alaska, U.S.A). (PDF)

S3 File Nei’s genetic distance (below diagonal) and *F*_ST_ (above diagonal, significant values in bold) for pairs of geographically separated haploid *S*. *magellanicum* groups. (PDF)

S4 File List of GenBank accession numbers for nucleotide sequences of *Sphagnum magellanicum* and *S*. *alaskense*. (PDF)
