# Supplementary material for: Spatial Genetic Structure of the Abundant and Widespread Peatmoss Sphagnum magellanicum Brid
Source: PLoS One. 2016 Feb 9;11(2):e0148447. doi: 10.1371/journal.pone.0148447 (PMC4747574; doi:10.1371/journal.pone.0148447)
Supplement: S1 File — (PDF) [file pone.0148447.s001.pdf]

DNA no.	Species	Locality	Herbarium	Herb ID
502	Sphagnum alaskense	USA:Alaska	DUKE	178856
503	Sphagnum alaskense	USA:Alaska	DUKE	131364
504	Sphagnum alaskense	USA:Alaska	DUKE	131365
505	Sphagnum alaskense	USA:Alaska	DUKE	131366
506	Sphagnum alaskense	USA:Alaska	DUKE	131367
507	Sphagnum alaskense	USA:Alaska	DUKE	131368
508	Sphagnum alaskense	USA:Alaska	DUKE	131369
509	Sphagnum alaskense	USA:Alaska	DUKE	126445
510	Sphagnum alaskense	USA:Alaska	DUKE	126481
511	Sphagnum alaskense	USA:Alaska	DUKE	81050
512	Sphagnum alaskense	USA:Alaska	DUKE	81340
513	Sphagnum alaskense	USA:Alaska	DUKE	130393
516	Sphagnum alaskense	Canada:British Columbia	DUKE	177565
517	Sphagnum alaskense	Canada:British Columbia	DUKE	177723
518	Sphagnum alaskense	USA:Alaska	DUKE	177566
585	Sphagnum alaskense	USA:Alaska	DUKE	
590	Sphagnum alaskense	USA:Alaska	TRH	724871
592	Sphagnum alaskense	USA:Alaska	TRH	726509
594	Sphagnum alaskense	USA:Alaska	TRH	726513
603	Sphagnum alaskense	USA:Alaska	TRH	726516
605	Sphagnum alaskense	USA:Alaska	TRH	726536
612	Sphagnum alaskense	USA:Alaska	TRH	726531
121	Sphagnum magellanicum	Canada:British Columbia	TRH	724881
122	Sphagnum magellanicum	Canada:British Columbia	TRH	724882
132	Sphagnum magellanicum	USA:Alaska	TRH	724871
134	Sphagnum magellanicum	USA:Alaska	TRH	724872
166	Sphagnum magellanicum	China:Heilongjiang	TRH	727476
172	Sphagnum magellanicum	China:Heilongjiang	TRH	741800
173	Sphagnum magellanicum	China:Jilin	TRH	741799
174	Sphagnum magellanicum	China:Jilin	TRH	741798
296	Sphagnum magellanicum	Russia:Amur	LE	
297	Sphagnum magellanicum	Russia:Amur	LE	
301	Sphagnum magellanicum	Russia:Kamchatka	LE	
302	Sphagnum magellanicum	Russia:Khabarovsk	LE	
305	Sphagnum magellanicum	Russia:Khabarovsk	LE	
306	Sphagnum magellanicum	Russia:Khabarovsk	LE	
307	Sphagnum magellanicum	Russia:Khabarovsk	LE	
314	Sphagnum magellanicum	Russia:Chukotka	LE	
316	Sphagnum magellanicum	Russia:Chukotka	LE	
317	Sphagnum magellanicum	Russia:Chukotka	LE	
318	Sphagnum magellanicum	Russia:Chukotka	LE	
321	Sphagnum magellanicum	Russia:Yamal	LE	
323	Sphagnum magellanicum	Russia:Yamal	LE	
329	Sphagnum magellanicum	USA:Alaska	LE	
336	Sphagnum magellanicum	USA:Alaska	LE	

337	Sphagnum magellanicum	Russia:Sachalin	MHA	
338	Sphagnum magellanicum	Russia:Sachalin	MHA	
339	Sphagnum magellanicum	Russia:Sachalin	MHA	
340	Sphagnum magellanicum	Russia:Yakutia	MHA	
341	Sphagnum magellanicum	Russia:Yakutia	MHA	
343	Sphagnum magellanicum	Russia:Khabarovsk	MHA	
344	Sphagnum magellanicum	Russia:Khabarovsk	MHA	
346	Sphagnum magellanicum	Russia:Yakutia	MHA	
519	Sphagnum magellanicum	USA:Alaska	DUKE	85298
520	Sphagnum magellanicum	USA:Alaska	DUKE	85296
521	Sphagnum magellanicum	USA:Alaska	DUKE	85318
522	Sphagnum magellanicum	USA:Alaska	DUKE	85316
523	Sphagnum magellanicum	USA:Alaska	DUKE	85314
524	Sphagnum magellanicum	USA:Alaska	DUKE	85326
525	Sphagnum magellanicum	USA:Alaska	DUKE	176665
526	Sphagnum magellanicum	USA:Alaska	DUKE	176855
527	Sphagnum magellanicum	USA:Alaska	DUKE	176841
528	Sphagnum magellanicum	USA:Alaska	DUKE	177573
529	Sphagnum magellanicum	USA:Alaska	DUKE	105991
530	Sphagnum magellanicum	USA:Alaska	DUKE	12476
531	Sphagnum magellanicum	USA:Alaska	DUKE	21513
532	Sphagnum magellanicum	USA:Alaska	DUKE	81358
533	Sphagnum magellanicum	USA:Alaska	DUKE	6534
556	Sphagnum magellanicum	USA:Iowa	DUKE	174918
588	Sphagnum magellanicum	USA:Alaska	TRH	724872
589	Sphagnum magellanicum	USA:Alaska	TRH	724871
593	Sphagnum magellanicum	USA:Alaska	TRH	726511
595	Sphagnum magellanicum	USA:Alaska	TRH	726514
596	Sphagnum magellanicum	USA:Alaska	TRH	726515
597	Sphagnum magellanicum	USA:Alaska	TRH	726517
599	Sphagnum magellanicum	USA:Alaska	TRH	726518
600	Sphagnum magellanicum	USA:Alaska	TRH	726522
601	Sphagnum magellanicum	USA:Alaska	TRH	766522
604	Sphagnum magellanicum	USA:Alaska	TRH	726519
606	Sphagnum magellanicum	USA:Alaska	TRH	726530
609	Sphagnum magellanicum	USA:Alaska	TRH	726528
610	Sphagnum magellanicum	USA:Alaska	TRH	726533
611	Sphagnum magellanicum	USA:Alaska	TRH	726531
1296	Sphagnum magellanicum	:	TRH	727477
106	Sphagnum magellanicum	Argentina:Tierra del Fuego	TRH	727050
107	Sphagnum magellanicum	Argentina:Tierra del Fuego	TRH	727451
109	Sphagnum magellanicum	Argentina:Tierra del Fuego	TRH	727449
110	Sphagnum magellanicum	Argentina:Tierra del Fuego	TRH	727452
111	Sphagnum magellanicum	Argentina:Tierra del Fuego	TRH	727457
113	Sphagnum magellanicum	Canada:Alberta	TRH	724817
116	Sphagnum magellanicum	Canada:British Columbia	TRH	724831

117	Sphagnum magellanicum	Canada:British Columbia	TRH	724826
118	Sphagnum magellanicum	Canada:British Columbia	TRH	724827
119	Sphagnum magellanicum	Canada:British Columbia	TRH	724828
120	Sphagnum magellanicum	Canada:British Columbia	TRH	724832
123	Sphagnum magellanicum	Canada:British Columbia	TRH	724884
125	Sphagnum magellanicum	Canada:Newfoundland and Labrador	TRH	724870
126	Sphagnum magellanicum	Canada:Newfoundland and Labrador	TRH	725163
127	Sphagnum magellanicum	Canada:Newfoundland and Labrador	TRH	724876
128	Sphagnum magellanicum	Canada:Newfoundland and Labrador	TRH	724877
129	Sphagnum magellanicum	Canada:Quebec	TRH	724878
133	Sphagnum magellanicum	Chile:	TRH	727462
135	Sphagnum magellanicum	USA:Alaska	TRH	724874
136	Sphagnum magellanicum	USA:Alaska	TRH	724863
137	Sphagnum magellanicum	USA:Connecticut	TRH	724851
138	Sphagnum magellanicum	USA:Florida	TRH	724868
139	Sphagnum magellanicum	Norway:Akershus	TRH	740633
140	Sphagnum magellanicum	Norway:Hedmark	TRH	740596
141	Sphagnum magellanicum	Norway:Hordaland	TRH	740658
144	Sphagnum magellanicum	Norway:Møre og Romsdal	TRH	740054
145	Sphagnum magellanicum	Norway:Nordland	TRH	157461
148	Sphagnum magellanicum	Norway:Nord-Trøndelag	TRH	740414
151	Sphagnum magellanicum	Norway:Østfold	TRH	10049
154	Sphagnum magellanicum	Norway:Sogn og Fjordane	TRH	740302
157	Sphagnum magellanicum	Norway:Sør-Trøndelag	TRH	158957
158	Sphagnum magellanicum	Norway:Oppland	TRH	VP149
159	Sphagnum magellanicum	Sweden:Halland	TRH	10010
160	Sphagnum magellanicum	Sweden:Värmland	TRH	10149
161	Sphagnum magellanicum	Denmark:Fyn	TRH	10125
162	Sphagnum magellanicum	Denmark:Nordjylland	TRH	10160
164	Sphagnum magellanicum	Denmark:Vestjylland	TRH	10189
165	Sphagnum magellanicum	Estonia:Pärnumaa	TRH	727419
168	Sphagnum magellanicum	Ecuador:Azuay	TRH	727467
171	Sphagnum magellanicum	Canada :Newfoundland and Labrador	TRH	741776
298	Sphagnum magellanicum	Chile:Chiloe	TRH	673507
300	Sphagnum magellanicum	Russia:Kamchatka	LE	
308	Sphagnum magellanicum	Russia:Tyumen	LE	
310	Sphagnum magellanicum	Russia:Irkutsk	LE	
312	Sphagnum magellanicum	Russia:Zabaikalski	LE	
319	Sphagnum magellanicum	Russia:Mari-El Republic	LE	
320	Sphagnum magellanicum	Georgia:Svaneti	LE	
324	Sphagnum magellanicum	Russia:Tyumen	LE	
325	Sphagnum magellanicum	Russia:Yamal	LE	
334	Sphagnum magellanicum	Canada:Ontario	LE	

342	Sphagnum magellanicum	Russia:Khabarovsk	MHA	
345	Sphagnum magellanicum	Russia:Tyumen	MHA	
347	Sphagnum magellanicum	Russia:Altai	MHA	
348	Sphagnum magellanicum	Russia:Altai	MHA	
349	Sphagnum magellanicum	Russia:Krasnoyarsk	MHA	
403	Sphagnum magellanicum	Scotland:Argyll and Bute	TRH	120111
404	Sphagnum magellanicum	Scotland:Argyll and Bute	TRH	120115
405	Sphagnum magellanicum	Scotland:Highland	TRH	120143
406	Sphagnum magellanicum	Scotland:Highland	TRH	120146
407	Sphagnum magellanicum	Northern Ireland:Fermanagh	TRH	120159
408	Sphagnum magellanicum	Ireland:Mayo	TRH	120165
409	Sphagnum magellanicum	Ireland:Galway	TRH	120170
410	Sphagnum magellanicum	Ireland:Roscommon	TRH	120172
411	Sphagnum magellanicum	Ireland:Meath	TRH	120175
552	Sphagnum magellanicum	USA:Maryland	DUKE	51813
553	Sphagnum magellanicum	USA:Maine	DUKE	179146
554	Sphagnum magellanicum	USA:Maine	DUKE	179156
555	Sphagnum magellanicum	USA:Kentucky	DUKE	51804
557	Sphagnum magellanicum	USA:Georgia	DUKE	51787
558	Sphagnum magellanicum	USA:Florida	DUKE	51766
559	Sphagnum magellanicum	USA:Conneticut	DUKE	13726
560	Sphagnum magellanicum	USA:Alabama	DUKE	68338
561	Sphagnum magellanicum	USA:Wisconsin	DUKE	11888
562	Sphagnum magellanicum	USA:West Virgina	DUKE	12733
563	Sphagnum magellanicum	USA:Virgina	DUKE	68514
564	Sphagnum magellanicum	USA:Vermont	DUKE	67848
565	Sphagnum magellanicum	USA:Tennessee	DUKE	51873
566	Sphagnum magellanicum	USA:Pennsylvania	DUKE	184679
567	Sphagnum magellanicum	USA:New York	DUKE	68298
568	Sphagnum magellanicum	USA:New Jersey	DUKE	68629
569	Sphagnum magellanicum	USA:New Hampshire	DUKE	67813
570	Sphagnum magellanicum	USA:Mississippi	DUKE	111375
571	Sphagnum magellanicum	USA:Minnesota	DUKE	199035
572	Sphagnum magellanicum	USA:Michigan	DUKE	85367
573	Sphagnum magellanicum	Japan:Hokkaido	TRH	727475
584	Sphagnum magellanicum	USA:Alaska	DUKE	Blanka
586	Sphagnum magellanicum	USA:North Carolina	TRH	120349
783	Sphagnum magellanicum	Germany:Baden-Wurttemberg	TRH	695137
790	Sphagnum magellanicum	Bolivia:La Paz	MA	27536
791	Sphagnum magellanicum	Bolivia:Cochabamba	MA	27923
792	Sphagnum magellanicum	Bolivia:Santa Cruz	MA	27924
793	Sphagnum magellanicum	Latvia:Sigulda	TRH	120182
794	Sphagnum magellanicum	Latvia:Krimuldas	TRH	120198
795	Sphagnum magellanicum	Latvia:Limbazu Apripkis	TRH	120216
797	Sphagnum magellanicum	Estonia:Viljandimaa	TRH	120242
798	Sphagnum magellanicum	Estonia:Jõgeva	TRH	120271

799	Sphagnum magellanicum	Estonia:Lääne-Virumaa	TRH	120290
800	Sphagnum magellanicum	Estonia:Ida-Virumaa	TRH	120300
801	Sphagnum magellanicum	Estonia:Harjumaa	TRH	120324
802	Sphagnum magellanicum	Estonia:Harjumaa	TRH	120338
815	Sphagnum magellanicum	Germany:Baden-Wurttemberg	TRH	695274
816	Sphagnum magellanicum	Norway:Sogn og Fjordane	TRH	742440
817	Sphagnum magellanicum	Norway:Sogn og Fjordane	TRH	742439
818	Sphagnum magellanicum	Norway:Sogn og Fjordane	TRH	742444
819	Sphagnum magellanicum	Norway:Sogn og Fjordane	TRH	742443
820	Sphagnum magellanicum	Norway:Sogn og Fjordane	TRH	742442
821	Sphagnum magellanicum	Norway:Sogn og Fjordane	TRH	742441
822	Sphagnum magellanicum	Denmark:Vestjylland	TRH	120426
1050	Sphagnum magellanicum	Russia: Kuril Islands	MHA	
