# Supplementary material for: Spatial Genetic Structure of the Abundant and Widespread Peatmoss Sphagnum magellanicum Brid
Source: PLoS One. 2016 Feb 9;11(2):e0148447. doi: 10.1371/journal.pone.0148447 (PMC4747574; doi:10.1371/journal.pone.0148447)
Supplement: S2 File — (PDF) [file pone.0148447.s002.pdf]

Continent		Collections	Haploid	Diploid	Misidentified	No DNA
S. magellanicum						
	Asia	53	15	25	0	13
	Europe	51	45	0	2	4
	Eastern North America	34	30	1	1	2
	Western North America	56	10	36	2	8
	South America	26	11	0	0	15
	Total	220	111	62	5	42
S. alaskense						
	Alaska	25	-	22	0	4
