# Supplementary material for: Spatial Genetic Structure of the Abundant and Widespread Peatmoss Sphagnum magellanicum Brid
Source: PLoS One. 2016 Feb 9;11(2):e0148447. doi: 10.1371/journal.pone.0148447 (PMC4747574; doi:10.1371/journal.pone.0148447)
Supplement: S3 File — (PDF) [file pone.0148447.s003.pdf]

Region	Western NA	Asia	Central Russia	Europe	Eastern NA	South America
Western NA		0.05	**0.13**	**0.26**	**0.08**	**0.20**
Asia	0.16		0	**0.25**	**0.07**	**0.29**
Central Russia	0.19	0.04		**0.29**	**0.15**	**0.33**
Europe	0.55	0.43	0.44		**0.11**	**0.31**
Eastern NA	0.26	0.20	0.24	0.20		**0.17**
South America	0.43	0.56	0.54	0.72	0.48	

Abbreviation: NA-North America
