# Supplementary material for: Spatial Genetic Structure of the Abundant and Widespread Peatmoss Sphagnum magellanicum Brid
Source: PLoS One. 2016 Feb 9;11(2):e0148447. doi: 10.1371/journal.pone.0148447 (PMC4747574; doi:10.1371/journal.pone.0148447)
Supplement: S4 File — (PDF) [file pone.0148447.s004.pdf]

DNA no.	Specimen voucher	Accesion number *trn*G	Accesion number *trn*L
106	TRH:727050	KU183771	KU183741
107	TRH:727451	KU183772	KU183742
113	TRH:724817	KU183773	KU183743
125	TRH:724870	KU183774	KU183744
129	TRH:724878	KU183775	KU183745
133	TRH:727462	KU183776	-
135	TRH:724874	KU183777	-
136	TRH:724863	KU183778	KU183746
137	TRH:724851	KU183779	KU183747
138	TRH:724868	KU183780	KU183748
139	TRH:740633	KU183781	KU183749
140	TRH:740596	KU183782	KU183750
168	TRH:727467	KU183783	KU183751
172	TRH:741800	KU183784	KU183752
173	TRH:741799	KU183785	KU183753
298	TRH:673507	KU183786	KU183754
318	LE:	KU183787	KU183761
411	TRH:120175	KU183788	KU183755
502	DUKE:178856	-	KU183762
510	DUKE:126481	KU183789	KU183763
517	DUKE:177723	-	KU183764
523	DUKE:85314	KU183790	KU183756
555	DUKE:51804	KU183791	KU183757
557	DUKE:51787	KU183792	KU183765
560	DUKE:68338	-	KU183766
570	DUKE:111375	KU183793	KU183770
573	TRH:727475	KU183794	KU183767
588	TRH:724872	KU183795	KU183759
589	TRH:724871	KU183796	KU183758
612	TRH:726531	KU183797	KU183760
790	MA:27536	KU183798	KU183768
791	MA:27923	KU183799	KU183769
